# *Fasciola* spp. in Southeast Asia: a systematic review and meta-analysis protocol

**DOI:** 10.1186/s13643-022-02013-3

**Published:** 2022-07-05

**Authors:** Vinh Hoang Quang, Bruno Levecke, Dung Do Trung, Brecht Devleesschauwer, Binh Vu Thi Lam, Katja Polman, Steven Callens, Pierre Dorny, Veronique Dermauw

**Affiliations:** 1 Department of Parasitology, National Institute of Malariology, Parasitology and Entomology, Hanoi, Vietnam; 2 Department of Translational Physiology, Infectiology and Public Health, Ghent University, Ghent, Belgium; 3 Unit of Veterinary Helminthology, Department of Biomedical Sciences, Institute of Tropical Medicine, Antwerp, Belgium; 4 Department of Epidemiology and Public Health, Sciensano, Brussels, Belgium; 5 Unit of Medical Helminthology, Department of Biomedical Sciences, Institute of Tropical Medicine, Antwerp, Belgium; 6 Department of Internal Medicine and Pediatrics, Faculty of Medicine and Health Sciences, Ghent University, Ghent, Belgium

**Keywords:** *Fasciola*, Liver fluke, Public health, Human, Livestock, Snail, Plants

## Abstract

**Background:**

Fascioliasis is an emerging public health threat in a number of regions worldwide, including Southeast Asia. Up to now, a summary of current knowledge on the occurrence and the distribution in Southeast Asia is lacking. We therefore aim to gather recent information on the distribution and prevalence of and the associated risk factors for *Fasciola* spp. infections in humans, animals, and plant carriers in Southeast Asia.

**Methods:**

Bibliographic and gray literature databases as well as reference lists of important review articles will be searched for relevant records that are published between January 1, 2000, and the search date. The systematic review will be reported according to the Preferred Reporting Items for Systematic Reviews and Meta-Analyses (PRISMA) guidelines for reporting systematic reviews. The primary outcomes will be both the prevalence of *Fasciola* spp. in the human and animal hosts, and on plant carriers in Southeast Asia, and the risk factors for occurrence of *Fasciola* spp. Secondary outcomes are the prevalence of *Fasciola* spp. in subpopulations (e.g., children and patients visiting clinics), the mapping of different diagnostic tests used, and the occurrence of the different *Fasciola* spp. in the study region. A descriptive statistical analysis will be conducted, and a meta-analysis will be run to estimate the prevalence of human and animal fascioliasis respectively, in Southeast Asia.

**Discussion:**

This systematic review will summarize the current knowledge on the epidemiology of *Fasciola* spp. infections in Southeast Asia.

**Systematic review registration:**

This systematic review has been registered with the International Prospective Register of Systematic Reviews (PROSPERO), reference number: CRD42021261104.

**Supplementary Information:**

The online version contains supplementary material available at 10.1186/s13643-022-02013-3.

## Background

Fascioliasis is a parasitic disease caused by the zoonotic worm species *Fasciola hepatica* and *Fasciola gigantica* [7]. This disease affects the liver of mammalian hosts and is primarily known for its high burden and associated economic impact in livestock. The annual global losses in animal production due to fascioliasis are estimated to be 3.2 billion US$ [9]. During the last decades, fascioliasis has also become an emerging public health problem. Today, it is estimated that 2.4 million people are infected and that 180 million people are at risk in over 70 countries [3]. The most affected countries are Bolivia, Peru, Egypt, Iran, and Vietnam [22]. As a response to fascioliasis being a global threat to public health, the World Health Organization (WHO) recommends large-scale deworming programs to populations at risk in endemic countries [22]. However, due to the complexity of the transmission cycle, deworming programs targeting humans only may not be the most cost-effective strategy for sustainable fascioliasis control. In addition, the growing number of reports of poor efficacy of triclabendazole against fascioliasis arising from drug resistance [5] underlines the necessity of a transdisciplinary approach.

The transmission cycle of fascioliasis involves a final host (harbouring the adult worms), an intermediate host (in which the larval stages develop and multiply), and a carrier (to which the infectious stage is attached). Infected livestock and humans (final hosts) shed worm eggs with their feces/stool. Once in a favorable environment, i.e., fresh water sources, miracidia (larval stage) develop in the eggs. Then, the eggs hatch and the miracidia infect freshwater snails (intermediate hosts). In the snail tissue, the miracidia undergo several development stages (sporocysts and rediae) and various rounds of asexual multiplication. Subsequently, an exponential number of free-swimming cercariae (larval stage) leave the snail and encyst into metacercariae (infectious stage) on water plants (the carrier). Susceptible livestock and humans acquire the infection by ingesting these contaminated raw water plants. Larval stages will migrate through the liver where they will mature in the bile ducts into hermaphrodite adult worms and start producing eggs [8]. From the transmission cycle, it is clear that not only human deworming, but also snail control and livestock treatment are valid options for *Fasciola* control [6]. Given the multi-faceted nature of the disease, a single intervention may not be sufficient for sustainable *Fasciola* control. Rather, a one health approach, bringing together a variety of disciplines and sectors, would be required [6, 15].

Transmission models are widely used tools to gain insight into the epidemiology of diseases. These models mimic complex phenomena and allow to assess the impact of a large number of interventions in in silico laboratories at low cost; this would be impossible in field studies due to ethical restrictions, high operational costs, and limited time to observe the impact of the interventions [1, 20]. Transmission models have already been described for a variety of parasitic diseases of which the majority have been successfully used to investigate control strategies [2, 16, 19]. Despite the clear advantages and the obvious need for more evidence-based guidance in control programmes, there is no comprehensive transmission model for *Fasciola* to date. The current models only partially describe the transmission cycle, excluding humans as a potential final host [17] or they make transmission predictions based on climatic factors [11, 18], thereby largely ignoring the underlying transmission mechanisms.

A transmission model for *Fasciola* spp. that includes all hosts and the plant carrier, albeit essential for truly understanding the disease epidemiology and control, is lacking up to now. To parametrize a complete transmission model, estimates for prevalence and risk factors in all hosts and the plant carrier need to be identified. These insights will be of utmost importance to further build and parameterize a comprehensive disease transmission model, which will focus on Southeast Asia, a region where fascioliasis is an emerging public health problem.

The aim of this systematic review is to gather recent information on the distribution and prevalence of, and associated risk factors for *Fasciola* spp. infections in humans, animals and plant carriers in Southeast Asia. The output of this review will not only identify knowledge gaps, and hence fostering more targeted research, but will also be crucial for more accurate parametrization of disease models.

## Methods

A systematic review on *Fasciola* spp. in Southeast Asia will be conducted. The review protocol is being reported in line with the Preferred Reporting Items for Systematic Reviews and Meta-Analyses Protocols (PRISMA-P) statement [10] (see Additional file 1), and the planned systematic review will be reported according to the Preferred Reporting Items for Systematic Reviews and Meta-Analyses (PRISMA) guidelines for reporting systematic reviews [12]. This systematic review has been registered with the International Prospective Register of Systematic Reviews (PROSPERO), reference number: CRD42021261104.

### Eligibility criteria

The systematic review will include all studies reporting data on *Fasciola* spp. in Southeast Asia (Brunei, Cambodia, Laos, Indonesia, Malaysia, Myanmar, Philippines, Singapore, Thailand, Timor-Leste, and Vietnam), published between January 1, 2000, and the search date. Records will be excluded based on the following criteria: (i) language not English; (ii) topic outside research question (i.e., not covering distribution and prevalence of and risk factors for *Fasciola* spp. in humans, animals, or plant carriers; (iii) data from outside the study region; (iv) data published beyond the study period; (v) no full-text available; or (vi) duplicate record.

### Information sources

Records will be retrieved from the bibliographic databases CINAHL, EMBASE, PubMed, Scopus, and Web of Science (all databases). Furthermore, gray literature will be sought from the following sources: Asian Digital Library (http://www.theadl.com), Google (https://google.com/), Google Scholar (https://scholar.google.com/), the Index Medicus for South-East Asian Region (https://www.globalindexmedicus.net/biblioteca/imsear/), and WHO IRIS (http://apps.who.int/iris/). Finally, reference lists of important review articles will be screened for relevant records.

### Search strategy

In the bibliographic databases, the following search phrase will be used, for the review period between January 1, 2000, and April 30, 2022: (fasciola OR fascioliasis OR fasciolosis OR *F. hepatica* OR *F. gigantica* OR liver fluke) AND (Southeast Asia OR Vietnam OR Laos OR Cambodia OR Indonesia OR Philippines OR Thailand OR Myanmar OR Malaysia OR Singapore OR Timor-Leste OR Brunei). This search phrase will be translated as follows for use in PubMed: (fasciol* OR “*F. hepatica*” OR “*F. gigantica*” OR “liver fluke”) AND (“Asia, Southeastern” [Mesh] OR “South East Asia” OR “Southeast Asia” OR “Southeastern Asia” OR Vietnam OR Laos OR Cambodia OR Indonesia OR Philippines OR Thailand OR Myanmar OR Malaysia OR Singapore OR Timor-Leste OR Brunei). For CINAHL, EMBASE, Scopus, and Web of Science (all databases), the search phrase will be translated as follows: (fasciol* OR “*F. hepatica*” OR “*F. gigantica*” OR “liver fluke”) AND (“South East Asia” OR “Southeast Asia” OR “Southeastern Asia” OR Vietnam OR Laos OR Cambodia OR Indonesia OR Philippines OR Thailand OR Myanmar OR Malaysia OR Singapore OR Timor-Leste OR Brunei).

The Asian Digital Library, Index Medicus for South-East Asian Region and WHO IRIS, databases will be searched for relevant hits using the search phrase: *Fasciola* OR fascioliasis OR fasciolosis OR liver fluke. Google and Google Scholar will be searched using the search phrase: (fasciola OR fascioliasis OR fasciolosis OR *F. hepatica* OR *F. gigantica* OR liver fluke) AND (Southeast Asia OR Vietnam OR Laos OR Cambodia OR Indonesia OR Philippines OR Thailand OR Myanmar OR Malaysia OR Singapore OR Timor-Leste OR Brunei). Additional records will be retrieved through screening of reference lists for relevant review articles

### Study records

After merging lists of records retrieved, duplicates will be removed. Next, titles and abstracts will be screened for relevance. The two members of the review team (VHQ and VD) will screen the search results independently and apply the inclusion and exclusion criteria. Inconsistent results will be discussed until consensus is reached.

Finally, full-text articles will be evaluated, and data are extracted from the retained records (see Data Items). Data collection forms will be designed and piloted before use. One member of the review team (VHQ) will extract the data. The data extraction will be subsequently be checked by a second member of the team (VD). Inconsistent results will be discussed until consensus is reached.

### Data items

From each retained record, author, reference, and publication year will be extracted. For population studies on humans, data for the following variables will be extracted: country, population studied, study period, number of people tested, number of positive individuals, *Fasciola* spp. detected, prevalence, and diagnostic test used. Additionally, for cross-sectional studies and cohort studies investigating risk factors, data for the following variables will be extracted: number of individuals with and without exposure of interest, and number of positive individuals in both groups. For case-control studies investigating the risk factors, number of cases and controls and number of individuals with exposure of interest in both groups will also be noted. For case reports, country and study period will be extracted. In case no contingency table is presented for these studies, univariate and multivariable odds ratios and associated 95% confidence intervals will be extracted as well.

For population studies on animals, data on the following variables will be extracted: country, population studied, study period, animal spp. tested, number of animals tested, number of positive individuals, *Fasciola* spp. detected, prevalence, and diagnostic test used. Additionally, the same variables as mentioned for cross-sectional, cohort, and case-control studies as well as case reports focussing on humans will be extracted for studies on animals. Both for human and animal intervention studies, baseline data (i.e., pre-intervention data) will be gathered.

For studies investigating carrier plants, data on the following variables will be extracted: country, study period, plant spp. tested, number of plant samples tested, number of positive samples, *Fasciola* spp. detected, prevalence, and diagnostic test used.

### Outcomes and prioritization

The primary outcome will be the prevalence of *Fasciola* spp. in the human and animal host and plant carriers in Southeast Asia. Moreover, the identified risk factors for occurrence of *Fasciola* spp. are considered as primary outcomes of this systematic review. Secondary outcomes are the prevalence of *Fasciola* spp. in subpopulations (e.g., children and patients visiting clinics), the mapping of different diagnostic tests used, and the occurrence of the different *Fasciola* species in the study region.

### Risk of bias in individual studies

The study quality of population studies will be assessed by the (modified) Newcastle–Ottawa scale [13, 21].

### Data synthesis

For the population studies, a descriptive statistical analysis will be conducted. For cross-sectional and cohort studies, the prevalence of *Fasciola* spp. will be calculated, and the associated Wilson score 95% confidence intervals will be calculated. Chi-square tests will be run to investigate the association between risk factors and presence of disease; in case of cell counts below 5, Fisher’s exact tests will be conducted. Odds ratios for the risk factors will be calculated as well as associated Wilson score 95% confidence intervals. The significance will be set at the 0.05 level. In case such calculations are not possible due to the lack of raw data, the reported summary statistics will be presented.

Next, a meta-analysis will be run to estimate the prevalence of human and animal fascioliasis respectively in Southeast Asia. For the human prevalence meta-analysis, only cross-sectional studies and baseline data from cohort or intervention studies will be included. Subjects should be a representative sample from the whole population in the study area, and reporting prevalence estimates should be based on stool microscopy. For the animal prevalence meta-analysis, the same criteria will be used, and additionally, only studies that report animal-level prevalence estimates will be included. The animal prevalence meta-analysis will be conducted per animal species. The percentage of variation across studies due to heterogeneity will be assessed using the Higgins *I*2 test statistic. To account for study heterogeneity, a random effects model will be used. A sensitivity analysis will investigate the impact of study design on the meta-analysis result. All statistical analyses will be carried out using the latest version of R [14].

### Meta-bias(es)

The presence of reporting bias will be explored by funnel plots.

### Confidence in cumulative evidence

The discussion of study results will include a description of the strengths and weaknesses of the studies. Our aim is to also include a summary of the quality of evidence, using the Grading of Recommendations, Assessments, Development and Evaluation (GRADE) approach [4].

## Discussion

Fascioliasis continues to be a neglected tropical disease, despite it being a well-known condition in livestock for decades. The recent emergence of fascioliasis in a number of regions, including Southeast Asia, and the complexity of the life cycle of the worm causing it, necessitate the evaluation of the most appropriate intervention strategies to stop transmission. The aim of this systematic review will be to gather current knowledge about *Fasciola* spp. infections in humans, animals, and plant carriers in Southeast Asia. It will summarize and describe estimates for the prevalence and risk factors in all hosts and the plant carrier. As such, the applications of this review will be two-fold. First, it will identify knowledge gaps and hence fostering more targeted research. Second, it will provide data that can be fed into a transmission model, which will ultimately assist policy makers in making evidence-based decisions about intervention strategies.

In the current review, only studies in English will be included. For some countries, this might lead to the introduction of language bias. Nevertheless, we expect that in the last 20 years, publishing in English language journals has become more common in the countries included in the study region. Moreover, we consider this systematic review as a first step in summarizing current knowledge on *Fasciola* spp. infections in humans, animals, and plant carriers in Southeast Asia. Once more data become available, additional, and updated estimates could be added to the current body of evidence on the prevalence and distribution of fascioliasis in Southeast Asia. The final report of this systematic review will be published in a peer-reviewed journal.

## Supplementary Information

**Additional file 1.**

## Data Availability

Not applicable.
